# Analysis of rice and wheat flour by particle nebulization ICP-MS

**DOI:** 10.1039/d0ra07224a

**Published:** 2020-11-26

**Authors:** Changzhi Shi, Wei Guo, Lanlan Jin, Shenghong Hu

**Affiliations:** State Key Laboratory of Biogeology and Environmental Geology, China University of Geosciences Wuhan 430074 P. R. China Wei.Guo@cug.edu.cn +86-27-67848602

## Abstract

Analysis of toxic elements in food samples (*e.g.*, rice and wheat) is very important for human health. A direct nebulization of solid particles for inductively coupled plasma (ICP) ionization and subsequent analysis of toxic elements (*i.e.*, As, Cd, Hg, and Pb) by mass spectrometry (MS) was developed. Dried and well-ground food particles (mean size of 0.9–1.0 μm) were stably dispersed in 0.5% polyethylene-imine (PEI) and the particle slurries were analyzed by ICP-MS using aqueous standard calibration. The transportation and ionization behaviors of particles with different particle sizes in ICP-MS were compared with those of aqueous standards containing equivalent concentrations of the analyte. The results indicated that the upper limits of particle sizes for the efficient transportation and complete ionization were 7.5–8.0 μm and 3.3–3.5 μm, respectively. Satisfactory recovery (94–107%), and precision (0.4–6.5%, RSD, *n* = 3) were verified by analyzing a series of rice and wheat standard reference materials (SRMs). The limits of quantitation (LOQs, 1.1 ng g^−1^ (Hg) to 3.5 ng g^−1^ (As)) are compared with the traditional microwave-assisted acid digestion ICP-MS method, however, the analysis throughput of the proposed method is improved by more than 10 times.

## Abstract 2

A simple and green particle nebulization ICP-MS method for the direct measurement of trace toxic elements in rice and wheat samples was developed.

## Graphical abstract

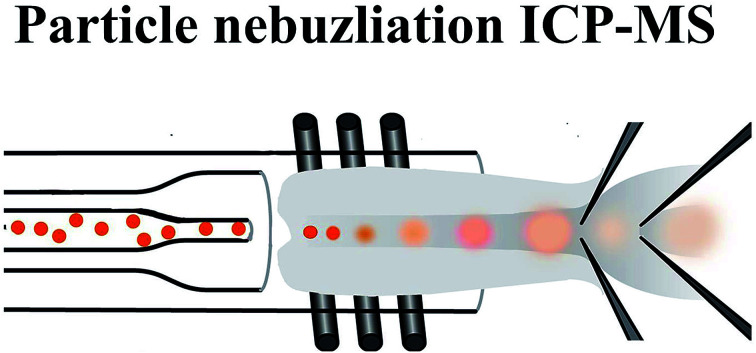

## Introduction

Rice and wheat are the two dominant staple foods for over half of the world's population, especially in Asian countries, contributing over 70% of the energy provided by their daily food intake. These are complex matrices made up of carbohydrates, proteins, fats, fiber and minor components of significant nutritional importance.^1,2^ On the other hand, these foods are also an important source of exposure to toxic elements (*i.e.*, As, Cd, Hg and Pb).^3^ The maximum toxic levels in rice and wheat established by the Chinese Government are 0.2 mg kg^−1^ (rice) and 0.5 mg kg^−1^ (wheat) for As, 0.2 mg kg^−1^ (rice) and 0.1 mg kg^−1^ (wheat) for Cd, 0.02 mg kg^−1^ (wheat or rice) for Hg and 0.2 mg kg^−1^ (wheat or rice) for Pb.^4^ Therefore, screening of these toxic elements in rice and wheat that are consumed is very important for human health.

Currently, the method based on inductively coupled plasma-mass spectrometry (ICP-MS) associated with microwave-assisted or closed vessel acid digestion has been considered the standard for the solid sample analysis,^5–13^ which due to its' distinct advantages, such as, reduction of the acid consumption, avoiding the losses of volatile elements (*i.e.*, B, Hg) and atmospheric contamination, high sensitivity and low detection limits, simultaneous multi-element and isotopic measurement.^14–21^ Although the microwave-assisted digestion ICP-MS method is widely used for the decomposition of various matrix samples, the pursuit of more green and simple analysis method without digestion procedure is still in progress. Laser ablation (LA)^22,23^ and slurry nebulization^24^ are two potential and attractive techniques by the direct introduction of sample particles or slurries into ICP-MS to complete simultaneously all the steps of matrix destruction, analyte atomization, excitation, ionization, and detection. The lack of available solid-matrix-matched external standards, which are required to overcome nonstoichiometric sampling, aerosol transport, and ionization in LA-ICP-MS analysis, remains a major drawback that hinders the accuracy and precision of this technique.^25^ Compared with the LA sampling technique, the advantage of slurry nebulization is that it has the potential for direct calibrations with conventional aqueous standards using a solution nebulization introduction system.^26^ Some applications of slurry nebulization ICP-optical emission spectrometry (ICP-OES) technique for the analysis of the geological and inorganic material samples are reported;^27–34^ however, the matrix effects in ICP-MS are much more serious than the ICP-OES technique. Recently, we have tried to use the slurry nebulization ICP-MS technique to measure the high field strength elements (Nb, Ta, Zr, and Hf) in silicate rocks.^35^ A Tissue Cell-Destroyer was firstly reported to produce ultra-fine particles (mean particle size, <1.0 μm) during 90 s of milling.^35^ Subsequently, this method has been applied to the determination of toxic elements and rare earth elements in tea.^36^ Satisfactory results show that this method has great potential to screening of multi-elements in tea with high fiber content.^36^ However, accurate quantitation calibration of the common foods (*i.e.*, rice and wheat) using the simple aqueous standards in particle nebulization ICP-MS technique remains a significant challenge and the ionization behaviors of particle slurries and aqueous standards should be also studied in detail.

In this work, the particle nebulization ICP-MS method for direct determination of the toxic elements (*i.e.* As, Cd, Hg, and Pb) in rice and wheat powders was investigated. Our research focuses on the direct calibrations with aqueous standards by studying the transportation and ionization behavior of particulate solid samples. The optimization of particle size reduction as well as the thorough validation of method performance by analyzing 10 certified reference materials (CRMs) are presented.

## Experimental

### Instrumentation, reagents, and standards

Particle nebulization analysis were conducted by a NexION 350D ICP-MS (PerkinElmer Ltd, Waltham, USA) with a conical U-series nebulizer (Glass Expansion Ltd, Port Melbourne Vic, Australia). High purity helium gas was used as the collision gas to eliminate the mass interferences in ICP-MS analysis. A tissue Cell-Destroyer 1000 (Hubei Xinzongke Viral Disease Control Bio-Tech Ltd, Wuhan, China) was used to achieve the desired particle sizes of the food samples by varying the milling time. The optimized operating parameters of ICP-MS and tissue Cell-Destroyer were listed in Table 1. Particle size distributions were measured by a Mastersizer 3000 laser diffraction analyzer (Malvern Panalytical Ltd, Malvern, England) and a S-4800 field emission scanning electron microscope (Hitachi Ltd, Tokyo, Japan). High-purity water (18.2 MΩ cm) obtained from a Millipore water purification system (Merck KGaA, Darmstadt, Germany) was used throughout for the preparation of samples and standard solutions. Standard solutions (1000 μg mL^−1^) of single elements and dispersant (polyethylene imine (PEI)) were obtained from the National Centre for Analysis and Testing of Steel Materials (Beijing, China) and Alfa Aesar Ltd (Tianjin, China), respectively. To evaluation the accuracy of the developed method, a series of rice and wheat standard reference materials (SRMs) (*e.g*. NIST 1567b wheat flour, NIST 1568c rice flour, GBW10011 wheat, GBW10035 wheat flour, GBW10046 wheat (Henan), GBW08503b wheat flour, GBW10010 rice, GBW10043 rice (Liaoning), GBW10043 rice (Sichuan), GBW10045 rice (Hunan) and GBW08502 rice flour) were purchased from the National Institute of Standards and Technology (Gaithersburg, MD) and the National Institute of Metrology (Beijing, China).

**Table 1:** Operation parameters for ICP-MS and particle size reduction

ICP-MS	PerkinEmer NexION 350D
RF power, W	1550
Plasma-gas flow, L min^−1^	17
Auxiliary-gas flow, L min^−1^	0.82
Nebulizer-gas flow, L min^−1^	0.91
KED He gas flow, mL min^−1^	4.0
Sampling depth, mm	−0.6
Sweeps	25
Reading per replicate	3
Dwell time, ms	50
Monitored ions	^75^As, ^111^Cd, ^202^Hg, and ^208^Pb
Internal standard ion	^103^Rh
Particle size reduction	Tissue Cell-Destroyer 1000
Rotation speed, rpm	5500
Each milling time, s	10
Time interval, s	10
Total milling time, s	120
Temperature, °C	37

### Measurement of the transportation and ionization behavior of particle slurries

To investigate the effect of slurry particle size on the transportation, ionization, and sensitivity of the method, the upper limit of the size of particles that can be transported into the ICP torch and the ionization efficiency in the ICP was investigated. The transportation behaviors of the slurry particles for wheat sample (NIST SRM 1567b) and rice sample (NIST SRM 1568b) were tested as follows: the original slurry with a mean particle size of 14.6 μm (wheat) and 15.3 μm (rice) was introduced into the nebulizer, and then the aerosol passed through the spray chamber and the torch injector, it was trapped in water and measured by Mastersizer 3000 analyzer.

The relative ionization efficiency (RIE) was involved to measure the effects of particle size on target ionization. The RIEs (%) for wheat sample (NIST SRM 1567b) and rice sample (NIST SRM 1568b) with different particle sizes were calculated using eqn (1):1
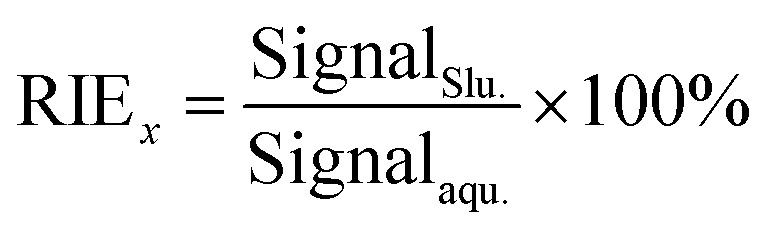


Signal_Slu._ is the signal intensity of As in counts per second (CPS) obtained by particle nebulization ICP-MS with a slurry concentration of 0.2%, Signal_aqu._ is the signal intensity of As (CPS) obtained by the conventional aqueous nebulization of a sample containing an equivalent concentration of the same element.

## Results and discussion

### Particle size reduction

Particle size reduction, followed by particle stabilization and homogenization are the two most important steps in sample preparation, and they significantly affect the analysis speed and result in particle-based techniques. After drying by heating oven, 0.10 g of wheat or rice SRM was ground using the tissue cell-destroyer in 1.5 mL 0.5% (m/v) PEI and 0.5 g zirconium oxide beads (o.d. = 0.15 mm). After milling using the optimized procedure (Table 1), the slurries containing particles were diluted to 50 mL with 0.5% (m/v) PEI and subsequently analyzed by ICP-MS.^36^ After 120 s of milling, slurries with mean particle sizes of 0.9–1.0 μm and 90% particles smaller than 1.8 μm were obtained for the rice or wheat SRMs (Table 2). The PEI was selected as the dispersing solvent because its long stability time (>2 h) compared with that of the other solvents. Above wet-grinding and solvent dispersion procedure integrates the rapid grinding of sample and the formation of stable and uniform particle slurries. It has a short time of sample preparation (120 s) than that of the traditional microwave-assisted acid digestion method, the analysis throughput has been improved by more than 10 times.

**Table 2:** Particle size distributions of wheat and rice materials (*n* = 3)

	Milling time	Mean diameter, μm	90% Particles, μm
NIST SRM SRM 1567b	10 s	7.0 ± 0.9	10.8 ± 1.1
	60 s	2.3 ± 0.5	4.9 ± 0.5
	120 s	0.9 ± 0.3	1.6 ± 0.4
	150 s	0.8 ± 0.2	1.4 ± 0.3
NIST SRM SRM 1568b	10 s	7.4 ± 0.9	11.6 ± 1.4
	60 s	2.5 ± 0.6	5.1 ± 0.8
	120 s	1.0 ± 0.3	1.8 ± 0.4
	150 s	0.9 ± 0.2	1.7 ± 0.3

### Behavior of particles transport and ionization

The prerequisite for accurate calibration using simple aqueous standards is that the analyte transportation and ionization efficiencies for the solid particles should be identical to those for the standard solution. Therefore, the transportation and ionization behaviors for particle nebulization and solution nebulization should be investigated. In initial experiment, the size distributions of food particles transported through the outlet of the torch injector of ICP-MS were determined (Table 3), the mean particle size of the rice and wheat materials collected from the torch outlet is smaller (4.5 μm for wheat and 4.6 μm for rice) than those in original slurries (14.6 μm for wheat and 15.4 μm for rice). The upper limits or cut-off sizes ranged from 8.0 (wheat) to 7.5 μm (rice), which are consistent with that of the tea materials (7.3 μm) in slurry nebulization ICP-MS analysis^36^ and the ceramic materials (7–10 μm) in slurry nebulization ICP-OES analysis.^37^ Particle ionization behavior was studied in terms of the RIE, which is used to normalize the response signal of analyte in particle nebulization to that in the aqueous standard containing an equivalent concentration of the same element (Experimental Section 2.2). As shown in Table 4, the upper size limit for particles that can be completely ionized (RIE > 95%) in ICP is 3.5 μm for wheat material and 3.3 μm for rice powder, respectively. The ionization efficiency for As decrease with increasing particle size, and only 32% of rice particles in size of 8.2 μm could be ionized. Compared with the coarse particles (>10 μm), the finer particles (<2.2 μm) are more easily transported to the ICP torch and are evaporated, atomized, and ionized in ICP plasma.

**Table 3:** Size distributions of wheat and rice particles in transportation process

Transport process	Wheat material (NIST 1567b)		Rice material (NIST 1568b)	
	Upper limit size^a^, μm	Mean diameter^b^, μm	Upper limit size^a^, μm	Mean diameter^b^, μm
Original slurry	18.7 ± 5.5	14.6 ± 3.2	19.4 ± 4.9	15.4 ± 3.8
Torch injector outlet	8.0 ± 1.1	4.5 ± 1.0	7.5 ± 1.2	4.6 ± 1.2

a The largest particle size was determined by FE-SEM.

b The particle mean diameter was determined by Mastersizer 3000 laser diffraction analyzer.

**Table 4:** The relative ionization efficiency (RIE) for wheat and rice materials in different particle sizes (*n* = 3)

	Particle size	Wheat material (NIST 1567b)	Rice material (NIST 1568b)
RIE^a^, %	0.8	100 ± 1.5	101 ± 2.0
	1.1	99.4 ± 1.8	100 ± 2.2
	2.2	98.6 ± 1.6	98.3 ± 2.4
	3.5	95.0 ± 2.1	94.3 ± 1.4
	5	88.6 ± 1.2	82.2 ± 1.6
	6.5	61.5 ± 1.6	53.5 ± 2.3
	8.2	35.3 ± 1.7	32.1 ± 2.4

a The RIE obtained by calculating the ratios of the signal intensity of As in particle slurry to those in aqueous solution containing equivalent concentration.

### Analytical performance

Calibration curves for standard aqueous solutions (adding 0.5% (m/v) PEI) with linear correlation coefficients (*R*^2^) >0.997 were constructed for these four toxic elements. The analytical performances were evaluated by analyzing a rice flour (NIST SRM 1568b) and a wheat flour (NIST SRM 1567b). Satisfactory recovery (94–107%), precision (0.4–6.5%, RSD, *n* = 3), and limits of quantitation (LOQs, 1.1 ng g^−1^ (Hg) to 3.5 ng g^−1^ (As)) are obtained (Table 5). The LOQs for the analytes were calculated as 11 times the standard deviation of 10 procedure blank measurements (taken using a blank solvent diluted 500 times), which are comparable with those for solution nebulization ICP-MS followed by microwave-assisted acid digestion method. In addition, analyte signal responses are stable (95–106%) during the running of 150 times for the rice or wheat SRMs, which indicated that the particles did not accumulate or block the interfaces in ICP-MS analysis.

**Table 5:** Analytical performance of the proposed particle nebulization ICP-MS method

		As	Cd	Hg	Pb
LOQ	This method (ng g^−1^)	3.5	2.5	1.1	3
	Microwave digestion ICP-MS (ng g^−1^)	3.0	2.0	2	2.1
NIST SRM 1567b	Measured values by this method (μg g^−1^)	4.61 ± 0.02	26.2 ± 0.5	0.48 ± 0.03	11.1 ± 0.28
	RSD, %	0.4	1.9	6.3	2.5
	Certified values (μg g^−1^)	4.8 ± 0.3	25.4 ± 0.9	0.5	10.4 ± 0.24
	Recovery, %	96	103	96	107
	Measured values by microwave digestion ICP-MS method (μg g^−1^)	4.72 ± 0.03	25.8 ± 0.4	0.47 ± 0.02	11.0 ± 0.12
NIST SRM 1568b	Measured values by this method (μg g^−1^)	268 ± 6	21.3 ± 0.2	5.67 ± 0.37	8.1 ± 0.3
	RSD, %	2.2	0.9	6.5	3.7
	Certified values (μg g^−1^)	285 ± 14	22.4 ± 1.3	5.91 ± 0.36	8 ± 3
	Recovery, %	94	95	96	101
	Measured values by microwave digestion ICP-MS method (μg g^−1^)	273 ± 5	21.7 ± 0.2	5.71 ± 0.26	8.0 ± 0.4

### Analysis of rice and wheat materials

The proposed particle nebulization ICP-MS method was used to analyze nine rice and wheat materials. Table 6 shows a comparison of the measured values with the reference values. The reference values were taken from the website of the China Geological Reference Materials Data Sharing Service System^38^ or were measured by the microwave-assisted acid digestion ICP-MS method. There are no differences (*P* < 0.05) in results obtained from the tested values by proposed method and reference values, further confirming the feasibility of the proposed particle nebulization technique for food analysis.

**Table 6:** Measured values of toxic elements in four wheat and five rice standard reference materials, *n* = 5, ng g^−1^

Materials	SRM no.	As		Cd		Hg		Pb	
		Measured values	Reference values	Measured values	Reference values	Measured values	Reference values	Measured values	Reference values
Wheat	GBW10011	30.5 ± 1.3	31 ± 5	17.4 ± 1.5	18 ± 4	1.5 ± 0.1	1.6	62.7 ± 3.2	65 ± 24
Wheat flour	GBW10035	24.2 ± 1.4	24.3 ± 1.7^a^	75.2 ± 2.1	74 ± 3	1.9 ± 0.2	1.8 ± 0.2^a^	1640 ± 15	1630 ± 30
Wheat (Henan)	GBW10046	26.2 ± 1.2	25	18.6 ± 1.3	18 ± 2	2.1 ± 0.1	2.2	65.3 ± 4.6	67 ± 16
Wheat flour	GBW08503b	325 ± 16	320 ± 70	150 ± 12	150 ± 40	2.3 ± 0.2	2.3 ± 0.3^a^	343 ± 10	340 ± 13
Rice	GBW10010	102 ± 4	102 ± 8	85.4 ± 2.3	87 ± 5	5.3 ± 0.2	5.4 ± 0.5	81.4 ± 4.2	80 ± 30
Rice (Liaoning)	GBW10043	117 ± 6	114 ± 18	12.5 ± 1.1	12 ± 3	4.9 ± 0.3	4.8 ± 0.8	75.3 ± 5.2	75 ± 25
Rice (Sichuan)	GBW10044	122 ± 8	120 ± 30	18.4 ± 1.3	18 ± 2	2.3 ± 0.2	2.2 ± 0.5	91.4 ± 6.4	90 ± 30
Rice (Hunan)	GBW10045	107 ± 11	110 ± 20	190 ± 14	190 ± 20	2.7 ± 0.2	2.8 ± 0.5	72.1 ± 6.8	70 ± 23
Rice flour	GBW08502	50.5 ± 2.4	51 ± 3	20.2 ± 1.2	20 ± 2	2.4 ± 0.3	2.4 ± 0.3^a^	748 ± 16	750 ± 50

a Reference values obtained by solution digestion ICP-MS method; other reference values obtained from the website of the China Geological Reference Materials Data Sharing Service System.^38^

## Conclusions

A simple and green particle nebulization ICP-MS method for the direct measurement of trace toxic elements in rice and wheat samples was developed. We found that the fine particle (∼1.0 μm) ensured efficient particle transportation and ionization and recoveries comparable with those of the standard solutions containing an equivalent concentration of the analyte. The proposed method has several advantages over conventional acid digestion ICP-MS technique including high throughput, easy to operate, low loss and contamination, and does not require a labor-intensive and time-consuming sample digestion procedure.

## Conflicts of interest

There are no conflicts to declare.

## Supplementary Material
